# Effects of Background Colors, Flashes, and Exposure Values on the Accuracy of a Smartphone-Based Pill Recognition System Using a Deep Convolutional Neural Network: Deep Learning and Experimental Approach

**DOI:** 10.2196/26000

**Published:** 2021-07-28

**Authors:** KyeongMin Cha, Hyun-Ki Woo, Dohyun Park, Dong Kyung Chang, Mira Kang

**Affiliations:** 1 Department of Digital Health Samsung Advanced Institute of Health Sciences & Technology Sungkyunkwan University Seoul Republic of Korea; 2 EvidNet Inc Seongnam-si, Gyeonggi-do Republic of Korea; 3 Division of Gastroenterology, Department of Internal Medicine Samsung Medical Center Sungkyunkwan University School of Medicine Seoul Republic of Korea; 4 Center for Health Promotion Samsung Medical Center Sungkyunkwan University School of Medicine Seoul Republic of Korea

**Keywords:** pill recognition, deep neural network, image processing, color space, color difference, pharmaceutical, imaging, photography, neural network, mobile phone

## Abstract

**Background:**

Pill image recognition systems are difficult to develop due to differences in pill color, which are influenced by external factors such as the illumination from and the presence of a flash.

**Objective:**

In this study, the differences in color between reference images and real-world images were measured to determine the accuracy of a pill recognition system under 12 real-world conditions (ie, different background colors, the presence and absence of a flash, and different exposure values [EVs]).

**Methods:**

We analyzed 19 medications with different features (ie, different colors, shapes, and dosages). The average color difference was calculated based on the color distance between a reference image and a real-world image.

**Results:**

For images with black backgrounds, as the EV decreased, the top-1 and top-5 accuracies increased independently of the presence of a flash. The top-5 accuracy for images with black backgrounds increased from 26.8% to 72.6% when the flash was on and increased from 29.5% to 76.8% when the flash was off as the EV decreased. However, the top-5 accuracy increased from 62.1% to 78.4% for images with white backgrounds when the flash was on. The best top-1 accuracy was 51.1% (white background; flash on; EV of +2.0). The best top-5 accuracy was 78.4% (white background; flash on; EV of 0).

**Conclusions:**

The accuracy generally increased as the color difference decreased, except for images with black backgrounds and an EV of −2.0. This study revealed that background colors, the presence of a flash, and EVs in real-world conditions are important factors that affect the performance of a pill recognition model.

## Introduction

Recently, smartphone cameras have been used to not only take photos but also recognize objects via models with enhanced performance and artificial intelligence models [1,2]. The study of photo recognition is not only limited to a person or a thing, such as a car; it can even extend to analyzing a person’s hair color or specifying the color of an object, such as a red car [3].

Many researchers are exploring new algorithms related to color in the field of image learning. For example, gray-scale images can be colored automatically by using a convolutional neural network (CNN) through a new method [4]. Additionally, Lunit—a well-known medical artificial intelligence company—presented an algorithm that enhances the color of an image as if it was corrected by a professional [5].

**Figure 1:**
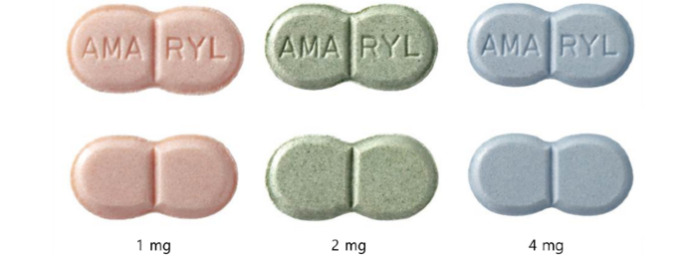
Examples of pills with the same physical features (except for color).

Color is an important component that is used to recognize objects, especially pharmaceuticals. The United States Federal Drug Administration approves solid pharmaceuticals and pills, which have physical identifiers. Each pill should have its own unique physical features, that is, unique shapes, sizes, colors, and imprints (the letter or number carved onto the medicine), which need to be approved [6]. However, in some cases, all features of medicines, except for the color, can be the same [7]. For instance, Amaryl (glimepiride)—an oral pill for controlling the blood sugar levels of patients with diabetes—has identical physical features across all 1-mg, 2-mg, and 4-mg dosages except for their colors (Figure 1).

**Figure 2:**
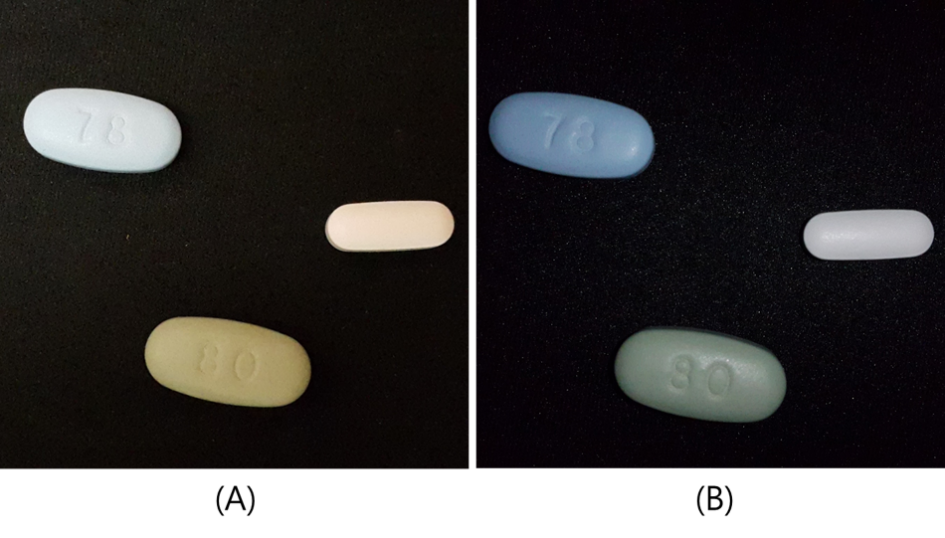
Effects that external environments (fluorescent lighting) have on the colors of pills in images. A: Flash is on. B: Flash is off.

In 2016, the National Institutes of Health hosted a competition to promote the easy recognition of unknown medications. Even though the competition used reference images that were photographed in a professionally supervised setting, the accuracy of drug recognition was not very high. Since the quality of a picture taken by a smartphone can be greatly influenced by illumination (lighting), shading, and background color, it is difficult to develop a system for image recognition [8]. Pill colors are especially affected by lighting hues and fluorescent light (Figure 2). In addition, there are no quantitative analyses for determining how a pill recognition system can be affected by external factors [6,9]. The most recent work related to drug recognition studies that involve deep learning has been conducted on wearable smart glasses developed for patients with visual impairment. Additionally, drug detection has been enhanced with feature pyramid networks and CNNs. However, despite recent improvements in pill recognition via a model approach, the effects of environmental factors have not been analyzed [10,11].

In this study, we sought to determine the accuracy of a pill recognition system under 12 different real-world conditions (ie, different background colors, the presence and absence of a flash, and different exposure values [EVs]).

## Methods

### Photo Shooting Equipment and Image Preprocessing

#### Data Acquisition Process for Reference Images

**Figure 3:**
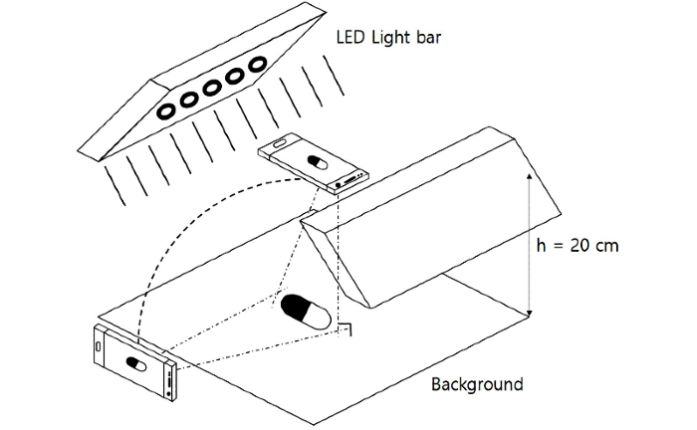
Photographic equipment (photo box) for taking images under the reference condition. LED: light-emitting diode.

The smartphone used in this study was the Samsung Galaxy S7 Edge, which was equipped with a dual-pixel 12.0-megapixel front camera with an aperture of f/1.7. An already intact camera app and the autofocus feature of the smartphone software were used. For lighting, 2 light-emitting diode panels were used. The flash was positioned at a height of 20 cm, and the intensity of illumination was set to 1145 lux. The background color was black, and the flash was turned off (Figure 3).

#### Data Acquisition Process for Real-World Images

The photos were taken under 12 conditions that involved different background colors (black or white), the presence or absence of a flash, and 3 different EVs (+2.0, 0, and −2.0; Table 1).

**Table 1:** Real-world image sets for the 12 conditions.

Image set name	Condition		
	Background color	Flash	Exposure value
B_O_EV-2.0	Black	On	−2.0
B_O_EV0	Black	On	0
B_O_EV+2.0	Black	On	+2.0
W_O_EV-2.0	White	On	−2.0
W_O_EV0	White	On	0
W_O_EV+2.0	White	On	+2.0
B_X_EV-2.0	Black	Off	−2.0
B_X_EV0	Black	Off	0
B_X_EV+2.0	Black	Off	+2.0
W_X_EV-2.0	White	Off	−2.0
W_X_EV0	White	Off	0
W_X_EV+2.0	White	Off	+2.0

#### Image Preprocessing

**Figure 4:**
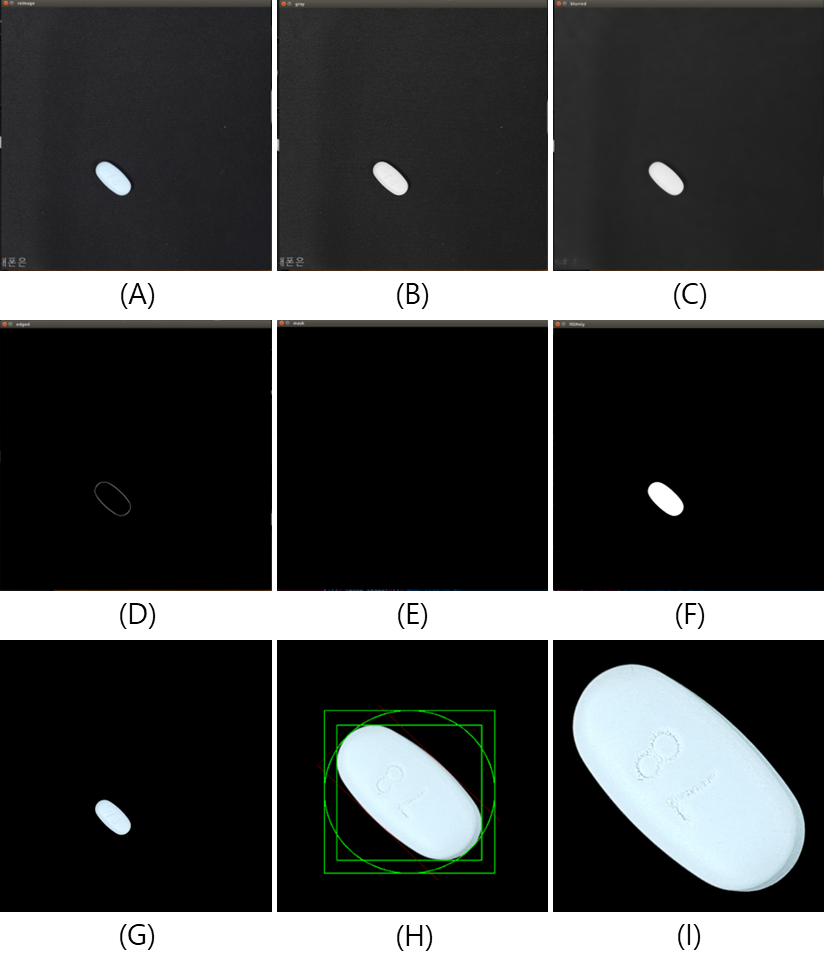
Image preprocessing algorithm for extracting an object. Step 1 (A): take pictures using a smartphone. Step 2 (B): convert image to a gray-scale image. Step 3 (C): use the blur and threshold options to process the image. Step 4 (D): Canny edge detection. Step 5 (E): create a black background. Step 6 (F): use the FillPoly function to process the image. Step 7 (G): use the bitwise operation to combine the original image with the processed image. Step 8 (H): draw a rectangle-shaped boundary and perform object extraction. Step 9 (I): use the final pill image as the reference image to train the model.

Figure 4 shows the 9 steps for processing images of the region of interest (ROI). This process was conducted to improve deep neural network–based image recognition accuracy by eliminating image noise and improving the quality of the picture [12]. Python 3.5.3 and the OpenCV 3.2 library were used to process each image [13]. The photos were converted to gray-scale images and blurred to reduce image noise. Afterward, we experimented with applying the different threshold options of the OpenCV library to each pill image. The Canny edge detector algorithm was used to define the ROI (a drug’s edge) [14,15]. Next, the processed picture was combined with the original picture, and all other areas except for the pill were omitted. Finally, the inner edge of the pill image was set within a square-shaped boundary, and this image was saved.

### Test Drug Type

**Figure 5:**
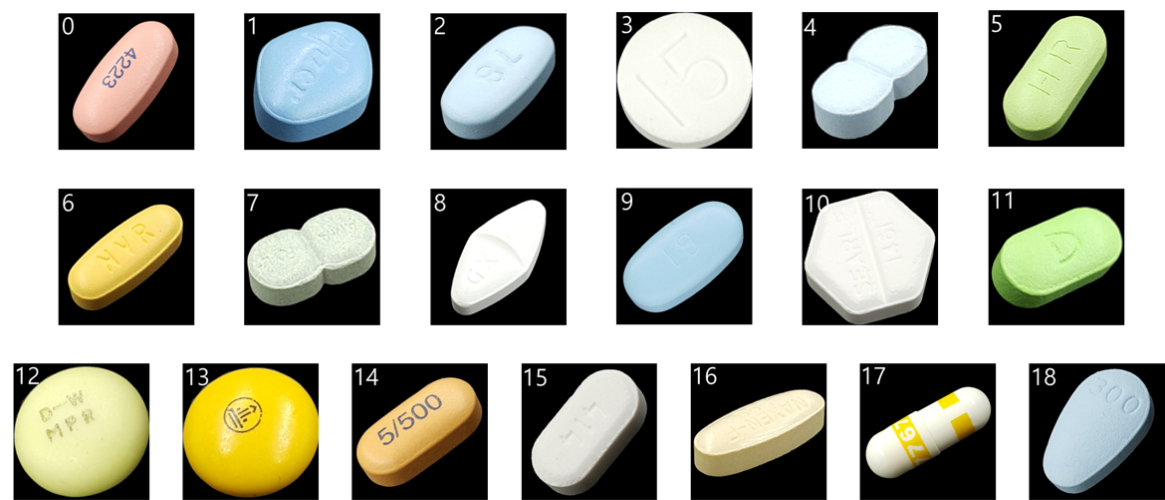
Sample images of the pills used in the experiment. Yellow pills include pills 6, 12, 13, 14, and 16. Green pills include pills 5, 7, and 11. “Other” pills include the rest of the pills.

A total of 19 different types of pills were used in this study. The different features of the pills (7 colors, 7 shapes, and 7 types) can be seen in Table 2. Figure 5 shows all of the example images of the pills; the numbers on the upper left-hand corners were the labels used in the deep learning process.

**Table 2:** Characteristics of the reference set (shape, color, and dosage form).

Characteristic		Instances, n
**Shape**		
	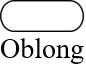	6
	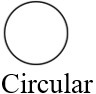	3
	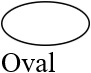	4
	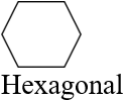	1
	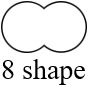	2
	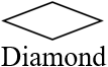	2
	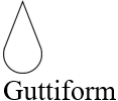	1
**Color**		
	Pink	1
	Blue	5
	White	5
	Yellow	4
	Green	1
	Yellow-green	2
	Orange	1
**Dosage form**		
	Film-coated tablet	10
	Sugar-coated tablet	2
	Uncoated tablet	6
	Hard capsule	1

### Color Difference

#### Factors Affecting the Color

**Figure 6:**
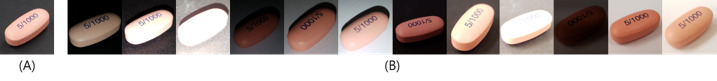
Representative examples of pill images. A: reference condition. B: 12 real-world conditions (image sets: B_O_EV-2.0, B_O_EV0, B_O_EV+2.0, W_O_EV-2.0, W_O_EV0, W_O_EV+2.0, B_X_EV-2.0, B_X_EV0, B_X_EV+2.0, W_X_EV-2.0, W_X_EV0, and W_X_EV+2.0).

Figure 6 shows the pill images that were taken under the reference condition and under the 12 real-world conditions. The colors of the pills differed based on the background colors, the presence of a flash, and EVs.

#### Color Space

The spatial color concept, which is expressed as a 3D chart, was used to calculate the differences in color quantitatively. The Commission Internationale de l’Eclairage (CIE) L*a*b* color space is a spatial color chart that is used worldwide to represent colors that can be detected by the human eye. After the red, green, and blue (RGB) color space is converted to a CIE XYZ color space, it is then converted to a CIE L*a*b* color space that separates the lighting and the color [16]. The CIE and CIE 1976 L*a*b* include some colors that human eyes cannot detect. L* represents brightness with values that range from 0 to 100. Parameters a* (green to red) and b* (blue to yellow) range from −120 to 120 [17,18].

**Figure 7:**
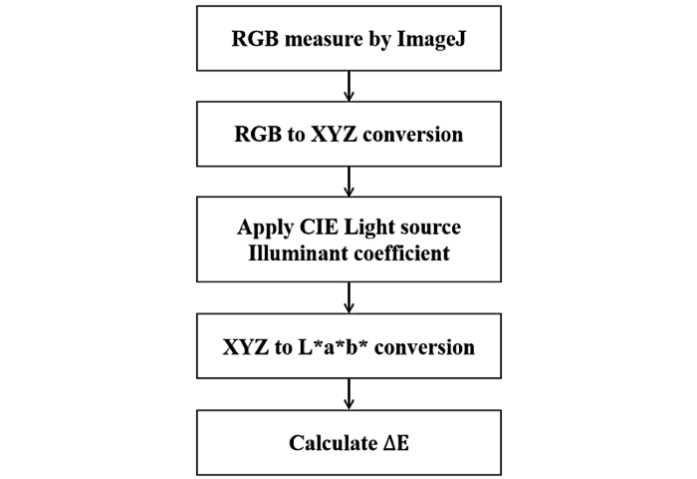
Color space conversion process. The conversion of RGB to CIE L*a*b* involves equations 1-8. △E is calculated by using equation 9. CIE: Commission Internationale de l’Eclairage; RGB: red, green, and blue.

To quantify the color of the ROI, the process shown in Figure 7 was followed. By using the RGB analysis plugin of the ImageJ 1.52 program (National Institutes of Health), the RGB color space was changed to an XYZ color space [19] via the following equations:

X = 0.4303R + 0.3416G + 0.1784B

**(1)**

Y = 0.2219R + 0.7068G + 0.0713B

**(2)**

Z = 0.0202R + 0.1296G + 0.9393B

**(3)**

The XYZ color space was then converted to an L*a*b* color space, as follows:

L* = 116f(Y/Y_n_) – 16

**(4)**

a* = 500(f[X/X_n_] – f[Y/Y_n_])

**(5)**

b* = 200(f[Y/Y_n_] − f(Z/Z_n_)]

**(6)**



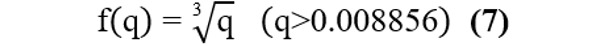

f(q) = 7.787q + (16/116) (q≤0.008856)

**(8)**

After computing the values of L*, a*, and b*, ∆E was calculated with the following equation, where ∆E is the color difference:



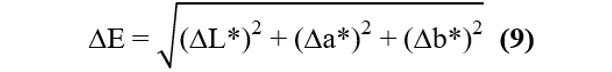



The color differences were calculated by subtracting the color distances in images taken under the real-world conditions from the color distances in images taken under the reference condition. The color distance of 19 medications was presented as means with SDs. A three-way repeated measures analysis of variance (ANOVA), which was followed by a Bonferroni posthoc test, was used to examine the effects that background color (black vs white), the presence of a flash (flash on vs flash off), and EV (+2.0, 0, and −2.0) had on color differences. A *P* value of <.05 was considered to be statistically significant. The statistical analysis was performed by using R software, version 3.6.2 (The R Foundation).

### Model Learning Process

A total of 34,000 images were taken manually by using a smartphone. We used images without augmentation. The number of images in the training set was 19,000, and the number of images in the validation set was 5000. We used 5000 images for the tests conducted under the reference condition and 5000 images for the tests conducted under real-world conditions.

The model architecture used in this study was a CNN that used a deep learning algorithm (GoogLeNet) with 22 layers and 9 inception modules [20]. We used the NVIDIA Deep Learning Graphics Processing Unit Training System (DIGITS) for the learning framework [21]. In this framework, top-1 accuracy refers to the extent to which a model’s answer exactly matches the expected answer. Top-5 accuracy refers to the extent to which the five highest model answers match the expected answer. Accuracy refers to the number of correct predictions divided by the total number of predictions. Loss refers to the penalty for a bad prediction. GoogLeNet has two auxiliary classifiers for combating the vanishing gradient problem. Loss1 is the first auxiliary classifier’s output, and Loss2 is the second auxiliary classifier’s output [20].

## Results

**Figure 8:**
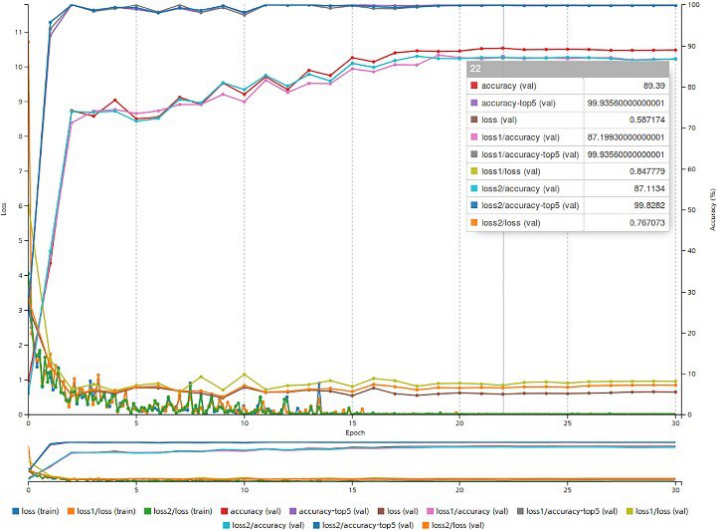
Model learning results. Top-1 accuracy refers to the extent to which a model’s answer exactly matches the expected answer. Top-5 accuracy refers to the extent to which the five highest model answers match the expected answer. Accuracy refers to the number of correct predictions divided by the total number of predictions. “(train)” refers to the training process and “(val)” refers to the validation process. Loss refers to the penalty for a bad prediction. Loss1 and Loss2 are two auxiliary classifiers of GoogLeNet.

**Figure 9:**
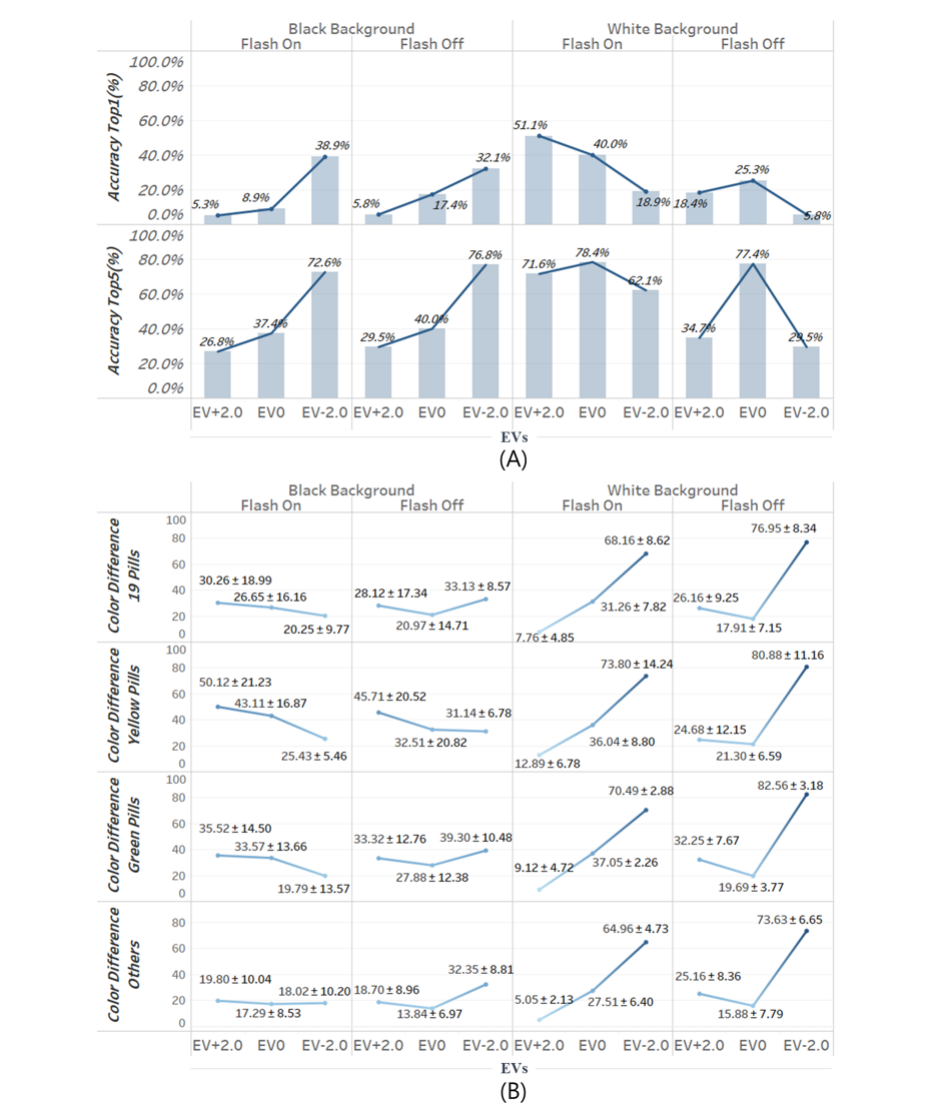
A: Comparison of top-1 and top-5 accuracies. B: Comparison of color differences based on background color, the presence of a flash, and EV. Color differences are presented as means with SDs. EV: exposure value.

Figure 8 shows the results of model training via the DIGITS framework. Our model recognized the correct pill with a top-1 accuracy of 84.54% and a top-5 accuracy of 99.89% for the reference test image set. Figure 9 shows the top-1 and top-5 accuracies and the average color differences for images taken under the 12 real-world conditions. For images with black backgrounds, as the EV decreased, the top-1 and top-5 accuracies increased independently of the presence of a flash. The top-5 accuracy for images with black backgrounds increased from 26.8% to 72.6% when the flash was on and increased from 29.5% to 76.8% when the flash was off as the EV decreased. However, the top-5 accuracy increased from 62.1% to 78.4% for images with white backgrounds when the flash was on. The best top-1 accuracy was 51.1% (white background; flash on; EV of +2.0). The best top-5 accuracy was 78.4% (white background; flash on; EV of 0). The results of the repeated measures ANOVA and the Bonferroni posthoc test for over 19 medications, as displayed in Figure 9, were used to assess the variances in color differences. Color differences based on EV values varied significantly (all *P* values in the repeated measures ANOVA were <.05). The results of the repeated measures ANOVA for color differences among 19 medications are as follows: *P*=.02 (black background and flash on); *P*=.02 (black background and flash off); *P*<.001 (white background and flash on); and *P<*.001 (white background and flash off). With regard to the Bonferroni posthoc test results, for images with white backgrounds that were taken with the flash turned on or off, all *P* values were <.001 between the image groups with different EVs. Color differences among images with black backgrounds that were taken with the flash turned on were statistically different between the EV +2.0 and EV 0 groups (*P=.*004) and between the EV0 and EV −2.0 groups (*P*=.004). Color differences among images with black backgrounds that were taken with the flash turned off were significantly different between the EV +2.0 and EV 0 groups (*P*=.005) and between the EV 0 and EV −2.0 groups (*P*=.03). When excluding the conditions of black backgrounds and an EV of −2.0, the accuracy generally increased as the color difference decreased. When the 19 medications were sorted into 3 groups by pill color (ie, yellow, green, and other), the color differences among the color subgroups were not dependent on the colors of pills in images with white backgrounds. However, the color differences among the color subgroups were dependent on the colors of pills in images with black backgrounds. The pill color, as well as environmental factors such as the background color, the presence of a flash, and EVs, can affect the accuracy of a pill recognition system (Figure 9).

## Discussion

The National Library of Medicine Pill Image Recognition Challenge was hosted by the National Institutes of Health in 2016. The three winners obtained a mean average precision of 0.27, 0.09, and 0.08. Their top-5 accuracy values were 43%, 12%, and 11% for 5000 query and consumer images. Although the competition can be seen as a promising initial step for pill identification, solid medication recognition systems are still in the difficult process of development. The reason for this seems to be that the quality of real-world images tends to be affected by illumination, shading, background color, or shooting direction, unlike reference images.

In our study, it was shown that smaller color differences yielded higher recognition accuracy except for images with black backgrounds and images with an EV of −2.0. In other words, the accuracy of pill recognition is generally inversely proportional to color difference. These exceptions may have been due to the following: (1) it is believed that the Euclidean distance between two colors may not be proportional to the precise color difference; and (2) other factors, such as pill imprints, shapes, and colors, can influence the recognition rate.

Color differences are a crucial problem, especially for pill recognition systems. In previous studies, a few methods were suggested for enhancing pill recognition. MedSnap (MedSnap LLC) is a smartphone-based pill identification system that uses an adaptive color correction algorithm. However, despite the fact that it corrects for color differences, this system has a disadvantage; it has to use a controlled surface to improve its pill recognition rate [22]. In a study on a deep learning model for dermatology, the authors recommended retaking the photo if it is of poor quality due to brightness or noise levels. Thus, adjusting the camera settings to match the optimized settings for a photo can yield better quality photos and improve the accuracy of medicine recognition systems [23,24]. Furthermore, the enhancement of drug detection via a model approach for minimizing color differences is warranted in the future.

This study reveals that background colors, the presence of a flash, and EVs in real-world conditions are important factors that affect the performance of pill recognition models. Depending on certain image conditions, pill colors can also affect pill recognition accuracy. However, this factor may not affect accuracy as much as environmental factors [25]. Further study is warranted on other factors, such as photography angles and heights, pill shapes, background colors, tablet and capsule conditions, and smartphone models that affect color differences and pill recognition accuracy [26-28].

## Abbreviations

ANOVA: analysis of variance
CIE: Commission Internationale de l’Eclairage
CNN: convolutional neural network
DIGITS: Deep Learning Graphics Processing Unit Training System
EV: exposure value
RGB: red, green, and blue
ROI: region of interest
